# Functional analysis of the global repressor Tup1 for maltose metabolism in *Saccharomyces cerevisiae*: different roles of the functional domains

**DOI:** 10.1186/s12934-017-0806-6

**Published:** 2017-11-09

**Authors:** Xue Lin, Ai-Qun Yu, Cui-Ying Zhang, Li Pi, Xiao-Wen Bai, Dong-Guang Xiao

**Affiliations:** 1 Tianjin Industrial Microbiology Key Laboratory, College of Biotechnology, Tianjin University of Science and Technology, Tianjin, 300457 People’s Republic of China; 2 College of Food Science and Technology, Hainan University, Haikou, 570228 China

**Keywords:** Baker’s yeast, Tup1, Functional domain, Maltose metabolism, Glucose repression

## Abstract

**Background:**

Tup1 is a general transcriptional repressor of diverse gene families coordinately controlled by glucose repression, mating type, and other mechanisms in *Saccharomyces cerevisiae*. Several functional domains of Tup1 have been identified, each of which has differing effects on transcriptional repression. In this study, we aim to investigate the role of Tup1 and its domains in maltose metabolism of industrial baker’s yeast. To this end, a battery of in-frame truncations in the *TUP1* gene coding region were performed in the industrial baker’s yeasts with different genetic background, and the maltose metabolism, leavening ability, *MAL* gene expression levels, and growth characteristics were investigated.

**Results:**

The results suggest that the *TUP1* gene is essential to maltose metabolism in industrial baker’s yeast. Importantly, different domains of Tup1 play different roles in glucose repression and maltose metabolism of industrial baker’s yeast cells. The Ssn6 interaction, N-terminal repression and C-terminal repression domains might play roles in the regulation of *MAL* transcription by Tup1 for maltose metabolism of baker’s yeast. The WD region lacking the first repeat could influence the regulation of maltose metabolism directly, rather than indirectly through glucose repression.

**Conclusions:**

These findings lay a foundation for the optimization of industrial baker’s yeast strains for accelerated maltose metabolism and facilitate future research on glucose repression in other sugar metabolism.

**Electronic supplementary material:**

The online version of this article (10.1186/s12934-017-0806-6) contains supplementary material, which is available to authorized users.

## Background

Maltose is the most abundant fermentable sugar in lean dough. The maltose metabolism level of baker’s yeast is the major factor for lean dough leavening ability [1]. The ability of a baker’s yeast strain to utilize maltose depends on one of the five unlinked *MAL* loci (*MAL1* through *MAL4* and *MAL6*) [2]. The typical locus *MAL6* contains three genes that are essential in metabolizing maltose: *MAL61* (*MAL6T*) encoding for maltose permease, *MAL62* (*MAL6S*) encoding for maltase, and *MAL63* (*MAL6R*) encoding for a positive regulatory protein, which activates the two enzymes [3]. Maltose is transported across the cell membrane via maltose permease, and cleaved intracellularly into two units of glucose by maltase. The synthesis of both enzymes is induced by maltose and repressed by glucose, and regulation occurs predominantly at the transcription level.

Transcriptional regulation by corepressors is an important mechanism for modulating the transcriptional activity of the genome [4]. Tup1–Ssn6, which consists of four Tup1 and one Ssn6 subunits, is one of the first corepressor complexes to be identified [5, 6]. According to the corepressor model, the Tup1–Ssn6 complex is recruited by pathway-specific DNA-binding proteins to mediate repression of different gene families during a wide variety of processes, such as glucose utilization, mating type, DNA damage repair and stress response [7]. It is largely accepted that Tup1 is the major contributor to the repression activity of the corepressor complex and Ssn6 acts as an adaptor in the complex [8–10]. Tup1 from *Saccharomyces cerevisiae* is a 713 amino acid protein and has at least three protein–protein interaction domains: the first 72 amino acids, the C-terminal seven WD repeats (340–713 amino acids), and the middle region in between. The N-terminal region of 72 amino acids interacts with Ssn6p and is required for self-tetramerization [11]. Amino acids 73-385 form the repression domain and interact with the N-terminal regions of histones H3 and H4 [12]. The C-terminal WD region mediates protein–protein interactions [13]. Tzamarias and Struhl have suggested that there are two independent repression domains in the middle region of the protein, amino acids 73–200 and 288–389 [14]. Johnson and co-workers also pointed out that Tup1 has two repression domains, one in the N-terminal and the other in the C-terminal in a region overlapping with the first WD repeat [15]. Different domains of Tup1 have been reported to participate in the control of expression of different target genes. Overexpression of the N-terminal 200 amino acids of Tup1 can almost completely repress *SUC2*, *RNR2*, and *OLE1*, but act as a dominant negative with regard to *MFA2*; deletion of the C-terminal repression domain alleviates repression of all tested target genes [16]. Expression of the N-terminal short fragment of *TUP1* conferred resistance to 5-bromodeoxyuridine in *S. cerevisiae* [17]. Overexpression of the truncated form of *TUP1* improved growth and fermentation capacities of *S. cerevisiae* on galactose [18]. Our previous study showed that although Tup1 is a transcriptional repressor, complete deletion of *TUP1* was not beneficial for glucose derepression to facilitate the maltose metabolism of baker’s yeast in the tested conditions [19]. Therefore, it is essential to investigate the role of Tup1 and its domains in maltose metabolism of industrial baker’s yeast.

**Fig. 1:**
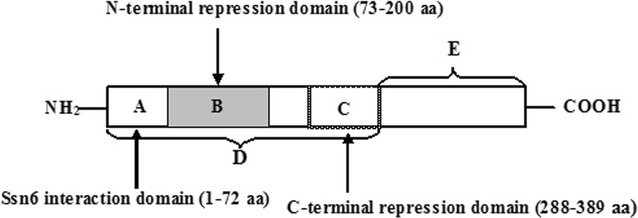
Structure of Tup1 functional domains. The different domains of Tup1 are depicted with A, B, C, D, E

In this study, a battery of in-frame (A through E domains in Fig. 1) truncations in the *TUP1* gene coding region were performed in the industrial baker’s yeasts with different genetic background. The maltose metabolism, leavening ability, *MAL* gene expression levels, and growth characteristics were measured to explore the role of Tup1 and its domains in maltose metabolism of industrial baker’s yeast.

## Methods

### Strains and vectors

The strains used in this study are summarized in Table 1.

**Table 1:** Strains used in the present study

Strains	Relevant characteristic	Reference or source
*Escherichia coli*		
DH5α	Φ80 *lacZ*ΔM15 Δ*lacU169 recA1 endA1 hsdR17 supE44 thi*-*1 gyrA relA1*	The Yeast Collection Center of the Tianjin Key Laboratory of Industrial Microbiology
*S. cerevisiae*		
BY14α	*MAT α*, Industrial baker’s yeast	The Yeast Collection Center of the Tianjin Key Laboratory of Industrial Microbiology
BYK	*MAT α*, Yep-K	This study
B + T	*MAT α*, Yep-KPT	This study
B + T_-C_	*MAT α*, Yep-KPT_-C_	This study
B + T_-B_	*MAT α*, Yep-KPT_-B_	This study
B + T_-A_	*MAT α*, Yep-KPT_-A_	This study
B + T_-D_	*MAT α*, Yep-KPT_-D_	This study
B + T_-E_	*MAT α*, Yep-KPT_-E_	This study
B-TUP1	*MAT α*, Δ*TUP1*:: *loxP*	The Yeast Collection Center of the Tianjin Key Laboratory of Industrial Microbiology
B-T + K	*MAT α*, Δ*TUP1*:: *loxP*, Yep-K	This study
B-T + T_-C_	*MAT α*, Δ*TUP1*:: *loxP*, Yep-KPT_-C_	This study
B-T + T_-B_	*MAT α*, Δ*TUP1*:: *loxP*, Yep-KPT_-B_	This study
B-T + T_-A_	*MAT α*, Δ*TUP1*:: *loxP*, Yep-KPT_-A_	This study
B-T + T_-D_	*MAT α*, Δ*TUP1*:: *loxP*, Yep-KPT_-D_	This study
B-T + T_-E_	*MAT α*, Δ*TUP1*:: *loxP*, Yep-KPT_-E_	This study

The vector pPGK1 was a generous gift from Professor Bauer F (Stellenbosch University, South Africa) [20]. The vectors Yep352 and pUG6 used in this study were purchased from Invitrogen (Carlsbad, Ca, USA).

### Growth, cultivation, and fermentation conditions

Recombinant plasmids were propagated in *Escherichia coli* DH5α, grown at 37 °C in Luria–Bertani medium (10 g/L tryptone, 5 g/L yeast extract, and 10 g/L NaCl) supplemented with 100 mg/L ampicillin. The plasmids were isolated using the Plasmid Mini Kit II (D6945, Omega, USA).

Yeast cells were grown to the stationary phase in yeast extract peptone dextrose (YEPD) medium (10 g/L yeast extract, 20 g/L peptone, and 20 g/L glucose). The precultures were inoculated into 200 mL of cane molasses medium at an initial OD_600_ of 0.4 and cultivated for 24 h at 30 °C with 180 rpm rotary shaking to a final OD_600_ of 1.8. Cells were harvested by centrifugation (4 °C, 1500×*g*, 5 min), and washed twice with cold sterile water for subsequent fermentation experiments. To investigate maltose metabolism of the strains, we used the low sugar model liquid dough (LSMLD) medium, which was modified from a medium described by Panadero et al. [21], and contains 2.5 g/L (NH_4_)_2_SO_4_, 5 g/L urea, 16 g/L KH_2_PO_4_, 5 g/L Na_2_HPO_4_, 0.6 g/L MgSO_4_, 0.0225 g/L nicotinic acid, 0.005 g/L Ca-pantothenate, 0.0025 g/L thiamine, 0.00125 g/L pyridoxine, 0.001 g/L riboflavin, and 0.0005 g/L folic acid and one of the two specified carbon sources (38 g/L maltose and 33.25 g/L maltose mixed with 5 g/L glucose).

### Construction of plasmid and yeast transformants

Yeast genomic DNA was prepared from the industrial baker’s yeast strain BY14α using the yeast DNA kit (D3370-01, Omega, USA). The PCR primers used in this study are listed in Table 2.

**Table 2:** Primers used in the present study (restriction sites are underlined)

Primer	Sequence (5′ → 3′)
For plasmid construction and verification	
TUP1-F	CCGCTCGAGATGACTGCCAGCGTTTCGAA
TUP1-R	CCGCTCGAGTTAATTTGGCGCTATTTTTTTA
PGK-F	CGCGCATGCTCTAACTGATCTATCCAAAACTGA
PGK-R	ACGCGCATGCTAACGAACGCAGAATTTTC
Kan-F	ACATGGATCCCAGCTGAAGCTTCGTACGC
Kan-R	ACATGGATCCGCATAGGCCACTAGTGGATCTG
T-F	CCGCTCGAGATGACTGCCAGCGTTTCGAATACG
T-R	CCGCTCGAGTTAATTTGGCGCTATTTTTTTATACTTCCAAATC
T_-C_-F	CTAGTTTGCAC CAGGATCACTACTTAGTCCTCATCGAAATTCGATCACTGAAAATAAC
T_-C_-R	ACGTGGTGGTGTTATTTTCAGTGATCGAATTTCGATGAGGGACTAAGTAGTGATCCGTG
T_-B_-F	CTAACTCACAGGAAAATGAAGGACGCGCCCCAGGTTTCCGTGGCA
T_-B_-R	AATGGTGCCACGGAAACCTGGGGCGCGTCCTTCATTTTCCTGTGA
T_-A_-F	CCGCTCGAGATGTACGAAGAAGAGATCAAGCACTTGAAAC
T_-A_-R	CCGCTCGAGTTAATTTGGCGCTATTTTTTTATACTTCCAAATC
T_-D_-F	CCGCTCGAGATGCATCGAAATTCGATCACTGAAAA
T_-D_-R	CCGCTCGAGTTAATTTGGCGCTATTTTTTTATAC
T_-E_-F	CCGCTCGAGATGACTGCCAGCGTTTCGAA
T_-E_-R	CCGCTCGAGTTAGTTATTGGCAGCAGAATCGTCAGA
For real-time PCR	
MAL62-F	AGTTTCCTGGCAAATCGG
MAL62-R	GTCCCACGGCAATCATAC
MAL61-F	TACCTCCGTTTGTTTGCG
MAL61-R	AGGACCATTGTGAGACCC
ACT1-F	ATTGATAACGGTTCTGGT
ACT1-R	AATTGGGTAACGTAAAGTC

Plasmid Yep-KPT (Yep-KanMX-PGK1-TUP1), an episomal plasmid containing *TUP1* under the control of the constitutive yeast phosphoglycerate kinase gene (*PGK1*) promoter (*PGK1*_*P*_) and terminator (*PGK1*_*T*_), was constructed as follow (graphically shown in Additional file 1: Figure S1): a *Bam*HI *KanMX* fragment, which was the dominant selection marker for yeast transformation, was amplified through PCR using pUG6 as the template with Kan-F and Kan-R primers and was cloned to the Yep352 vector to construct the empty plasmid Yep-K (Yep-KanMX). A *Xho*I fragment of *TUP1* amplified with TUP1-F and TUP1-R primers from genomic DNA of the parental strain BY14α was inserted downstream of the *PGK1p* of pPGK1 vector and resulted in the plasmid pPGKT. The *Sph*I fragment of PT (the entire *PGK1* with the inserted *TUP1*) amplified from pPGKT using primers PGK-F and PGK-R was cloned to Yep-K to generate the final plasmid Yep-KPT (the sequence shown in Additional file 2). Plasmid Yep-KPT_-A_ (Yep-KanMX-PGK1-TUP1_-A_) was constructed as above using T_-A_-F and T_-A_-R primers to amplify truncated *TUP1* lacking residues 1–72. Plasmid Yep-KPT_-D_ (Yep-KanMX-PGK1-TUP1_-D_) was constructed as above using T_-D_-F and T_-D_-R primers to amplify truncated *TUP1* lacking residues 1–389. Plasmid Yep-KPT_-E_ (Yep-KanMX-PGK1-TUP1_-E_) was constructed as above using T_-E_-F and T_-E_-R primers to amplify truncated *TUP1* lacking residues 390–713.

Plasmid Yep-KPT_-B_ (Yep-KanMX-PGK1-TUP1_-B_) was constructed in four steps as follow: (1) the upstream (T-NU) and downstream (T-ND) homologous sequences were amplified from genomic DNA of the parental strain BY14α using primers T-F/T_-B_-R and T-R/T_-B_-F, respectively; (2) after fusion PCR [3 cycles of 56 °C (15 s), 72 °C (90 s)], in which purified T-NU and T-ND fragments were used as templates without adding primer, the primers T-F and T-R were added to the product of the fusion PCR; (3) the resultant fusion PCR products purified by gel extraction were digested using *Xho*I and inserted into plasmid pPGK1 digested with the same enzyme, resulting the plasmid pPGKT_-B_; (4) the *Sph*I fragment of PT_-B_ (the entire *PGK1* and the inserted truncated *TUP1* lacking residues 73–200) amplified from pPGKT_-B_ using primers PGK-F and PGK-R was cloned to Yep-K to produce the final plasmid Yep-KPT_-B_. Plasmid Yep-KPT_-C_ (Yep-KanMX-PGK1-TUP1_-C_) was constructed as above using T-F/T_-C_-R and T-R/T_-C_-F primers for amplifying the homologous sequences.

Yeast transformation was performed using the lithium acetate/PEG procedure [22]. The YEPD plates were supplemented with 800 mg/L G418 for selection of the overexpression strains BYK, B + T, B + T_-C_, B + T_-B_, B + T_-A_, B + T_-D_, B + T_-E_, B-T + K, B-T + T_-C_, B-T + T_-B_, B-T + T_-A_, B-T + T_-D_ and B-T + T_-E_ after transformation. The transformants were then verified by PCR using the primers Kan-F/Kan-R, PGK-F/T-R, PGK-F/T_-C_-R, PGK-F/T_-B_-R, PGK-F/T_-A_-R, PGK-F/T_-D_-R, PGK-F/T_-E_-R, Kan-F/Kan-R, PGK-F/T_-C_-R, PGK-F/T_-B_-R, PGK-F/T_-A_-R, PGK-F/T_-D_-R, PGK-F/T_-E_-R, respectively.

### Analysis of sugar consumption

For extracellular sugar measurements, cultures were sampled at 30 °C at suitable intervals for 4 h. Samples were filtered through 0.45 μm pore size cellulose acetate filters (Millipore Corp, Danvers, MA, USA) and analysed by high-performance liquid chromatography (HPLC) using a refractive index detector and an Aminex^®^ HPX-87H column (Bio-Rad, Hercules, CA, USA) at 65 °C with 5 mM H_2_SO_4_ as the mobile phase at a flow rate of 0.6 mL/min [23].

The maltose utilization efficiency in maltose LSMLD medium was determined by the ratio of the consumed maltose in 240 min with the total maltose. The maltose utilization efficiency in maltose–glucose LSMLD medium was determined by the ratio of consumed maltose in 90 min, with the total maltose. The calculation formula of maltose utilization efficiency in maltose–glucose LSMLD medium was:

$${\text{Maltose utilization efficiency }}\left( \% \right) = [({\text{the content of total maltose}}-{\text{the content of residual maltose at }}90 \,\hbox{min} )/{\text{total maltose}}] \times 100\%$$

Based on the consumption curves of glucose and maltose in maltose-glucose LSMLD medium, the time span between the time point when half of the maltose had been consumed and when half of the glucose had been consumed was determined. Experiments were conducted thrice.

### Determination of leavening ability

The leavening ability of yeast cells in lean dough was determined according to the Chinese National Standards for yeast used in food processing. Leavening ability was determined by milliliter of CO_2_ production per hour per gram (dry weight) of yeast cells. Lean dough consisted of 280 g of flour, 150 mL of water, 4 g of salt, and 8 g of fresh yeast. The dough was evenly and quickly mixed for 5 min at 30 ± 0.2 °C, and placed inside the box of a fermentograph (Type JM451, Sweden). CO_2_ production was recorded at 30 °C. Experiments were conducted thrice.

### qRT-PCR

Total cellular RNA was extracted using a yeast RNA kit (Omega, Madison, United States). Using mRNA as template, cDNA was synthesized using a Reverse Transcription System Kit (Takara, China). The expression levels of *MAL62* and *MAL61* were assessed by real-time quantitative PCR (qRT-PCR) using an Ultra SYBR Two-Step qRT-PCR kit with ROX (reference dye for real-time PCR, TIANGEN, China) in two LSMLD media. Actin was used as a loading control. The primers used for amplifying target genes and reference gene *ACT1* are listed in Table 2. The expression level of the target genes in the strain BY14α was normalized to the reference gene. Experiments were conducted thrice.

### Determination of specific growth rate and biomass yield

After 24 h of incubation, the mixtures of cell culture and medium were mixed in a deep well plate in appropriate proportions, and the growth curve was formed using bioscreen automated growth curves (Type FP-1100-C, Oy Growth Curves Ab Ltd., Helsinki, Finland). The specific growth rate was determined with the change in the OD_600_ napierian logarithm versus the time during exponential growth.

Nitrocellulose filters with a pore size of 0.45 mm (Gelman Sciences, Ann Arbor, MI, USA) were pre-dried in a microwave oven at 150 W for 10 min and subsequently weighed. The cell culture (10 mL) in the stationary phase was filtered, washed twice with 10 mL of distilled water, and then dried at 105 °C for 24 h to measure the cell dry weight. The biomass yield was expressed as gram (dry weight) of yeast cells per liter of medium. Experiments were conducted thrice.

### Statistical analysis

Data were expressed as mean ± SD and were accompanied by the number of experiments independently performed. The differences of the transformants compared with the parental strain were confirmed by Student’s *t* test. Differences at *P* < 0.05 were considered significant differences in statistics.

## Results

### Sugar consumption of the transformants with gene fragments overexpressed in the parental strain BY14α in LSMLD media

**Fig. 2:**
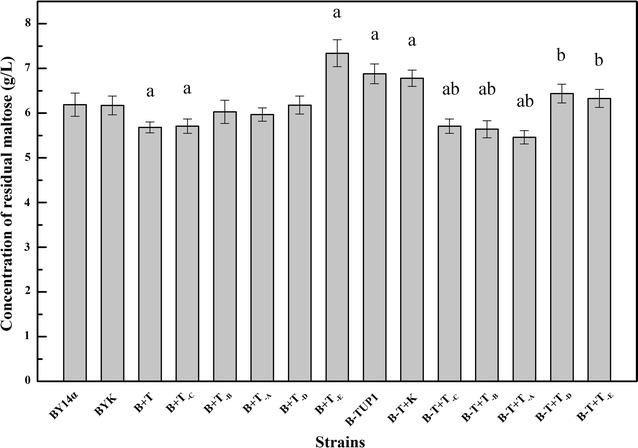
Concentration of residual maltose of the strains in maltose LSMLD medium. Data shown was sampled at 240 min. Data are average of three independent experiments and error bars represent ± SD. Significant differences of the strains (B + T, B + T_-C_, B + T_-E_, B-TUP1, B-T + K, B-T + T_-C_, B-T + T_-B_ and B-T + T_-A_) from the parental strain BY14α (^a^ *P* < 0.05, n = 3), and the strains (B-T + T_-C_, B-T + T_-B_, B-T + T_-A_, B-T + T_-D_ and B-T + T_-E_) from the mutant B-TUP1 (^b^ *P* < 0.05, n = 3) were confirmed by Student’s *t* test

**Fig. 3:**
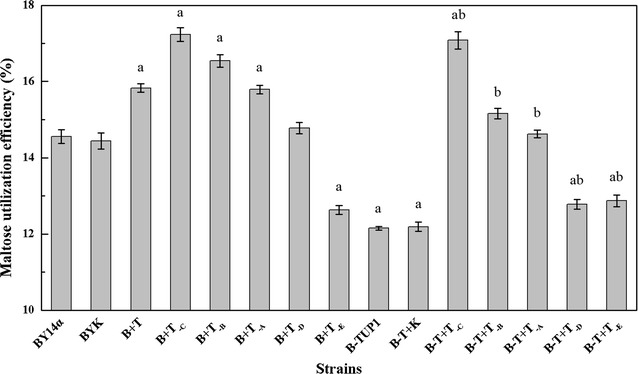
Maltose utilization efficiency of the strains in maltose-glucose LSMLD medium. Data are average of three independent experiments and error bars represent ± SD. Significant differences of the strains (B + T, B + T_-C_, B + T_-B_, B + T_-A_, B + T_-E_, B-TUP1, B-T + K, B-T + T_-C_, B-T + T_-D_ and B-T + T_-E_) from the parental strain BY14α (^a^ *P* < 0.05, n = 3), and the strains (B-T + T_-C_, B-T + T_-B_, B-T + T_-A_, B-T + T_-D_ and B-T + T_-E_) from the mutant B-TUP1 (^b^ *P* < 0.05, n = 3) were confirmed by Student’s *t* test

**Fig. 4:**
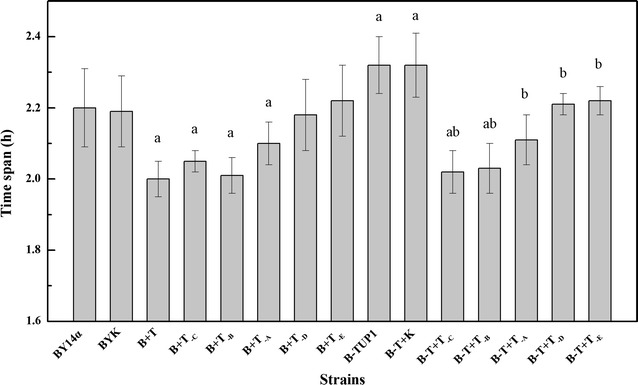
Time span of the strains. Data are average of three independent experiments and error bars represent ± SD. Significant differences of the strains (B + T, B + T_-C_, B + T_-B_, B + T_-A_, B-TUP1, B-T + K, B-T + T_-C_ and B-T + T_-B_) from the parental strain BY14α (^a^ *P* < 0.05, n = 3), and the strains (B-T + T_-C_, B-T + T_-B_, B-T + T_-A_, B-T + T_-D_ and B-T + T_-E_) from the mutant B-TUP1 (^b^ *P* < 0.05, n = 3) were confirmed by Student’s *t* test

In this study, the intact *TUP1* and various truncated forms of *TUP1* were overexpressed in the industrial baker’s yeast BY14α, and the sugar consumption of the strains was studied in LSMLD media. The strain BYK was used as a control to avoid any possible effects of the empty vector and exhibited similar sugar consumption to that of the parental strain BY14α (Figs. 2, 3). In maltose LSMLD medium, the residual maltose of the strains B + T, B + T_-C_, B + T_-B_ and B + T_-A_ was varying lower than that of the strain BY14α (Fig. 2, shown in Additional file 3: Table S1 ). In maltose-glucose LSMLD medium, the maltose utilization efficiencies of the strains B + T, B + T_-C_, B + T_-B_ and B + T_-A_ were 8.7, 18.3, 13.6 and 8.4% higher than that of the strain BY14α (Fig. 3). Simultaneously, the time span in the strains B + T, B + T_-C_, B + T_-B_ and B + T_-A_ decreased by 9.1, 6.8, 8.6 and 4.5%, respectively, compared with the parental strain (Fig. 4). For the E fragment overexpression strain B + T_-D_, negligible differences of sugar consumption were observed compared with the parental strain (Figs. 2, 3). However, 3.6 and 13.3% decreases in maltose utilization efficiency were observed in the strain B + T_-E_ in maltose and maltose-glucose LSMLD media, respectively (Figs. 2, 3). No obvious changes of the time span were observed in the strains B + T_-E_ and B + T_-D_ (Fig. 4).

These findings suggest that with the native copy of *TUP1* intact, overexpression of *TUP1* could lead to positive effect on glucose derepression with decreased time span, thereby promoting the maltose utilization of baker’s yeast. Furthermore, overexpressing variant *TUP1* with any of the three domains (Ssn6 interaction, N-terminal repression and C-terminal repression domain) truncated produced varying positive effects on glucose derepression and maltose utilization of baker’s yeast. Overexpression of *TUP1* with all three domains truncated did not influence glucose repression and maltose metabolism. However, overexpression of the three-domain fragment of *TUP1* was deleterious to maltose utilization of baker’s yeast.

### Sugar consumption of the transformants with gene fragments overexpressed in the *TUP1* deletion mutant B-TUP1 in LSMLD media

To further examine the effects of the different domains of Tup1 on maltose metabolism, truncated variants of *TUP1* were overexpressed in the *TUP1* deletion mutant B-TUP1, and the sugar consumption were investigated in LSMLD media. The control strain B-T + K exhibited similar sugar consumption to that of the mutant B-TUP1 (Figs. 2, 3). After 240 min of fermentation, the residual maltose in the strains B-T + T_-C_, B-T + T_-B_ and B-T + T_-A_ were obviously lower than that in the strain B-TUP1, and even lower than the parental strain BY14α in maltose LSMLD medium (Fig. 2, shown in Additional file 3: Table S1). In maltose-glucose LSMLD medium, 40.6, 24.8 and 20.3% increases in the maltose utilization efficiency were observed in the strains B-T + T_-C_, B-T + T_-B_ and B-T + T_-A_, respectively, and the time span decreased by 12.9, 12.5 and 9.1%, respectively, compared with the mutant B-TUP1 (Figs. 3, 4). Compared with the strain B-TUP1, B-T + T_-D_ and B-T + T_-E_ increased the maltose utilization efficiency by 5.2 and 5.9%, respectively, in maltose-glucose LSMLD medium, along with decreased time span, respectively, but the maltose utilization were still lower than those of the strain BY14α (Figs. 3, 4).

These results demonstrate that with *TUP1* deleted from the chromosome, overexpressing variant *TUP1* with any of the Ssn6 interaction, N-terminal repression and C-terminal repression domain truncated could significantly counter the glucose repression brought about by *TUP1* deletion and effectively enhance maltose metabolism of baker’s yeast. Although overexpressing *TUP1* with all three domains truncated or the three-domain fragment of *TUP1* could restore glucose repression to a certain degree, it is not enough to recover the delayed maltose metabolism.

### Fermentation characteristic

**Fig. 5:**
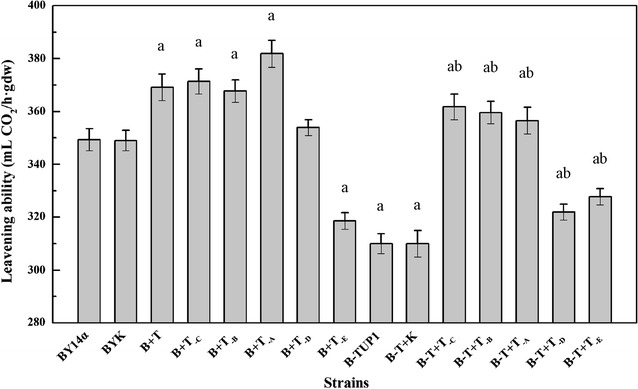
Leavening ability of the strains in lean dough. Data are average of three independent experiments and error bars represent ± SD. Significant differences of the strains (B + T, B + T_-C_, B + T_-B_, B + T_-A_, B + T_-E_, B-TUP1, B-T + K, B-T + T_-C_, B-T + T_-B_, B-T + T_-A_, B-T + T_-D_, B-T + T_-E_) from the parental strain BY14α (^a^ *P* < 0.05, n = 3), and the strains (B-T + T_-C_, B-T + T_-B_, B-T + T_-A_, B-T + T_-D_ and B-T + T_-E_) from the mutant B-TUP1 (^b^ *P* < 0.05, n = 3) were confirmed by Student’s *t* test

The leavening abilities of these 15 strains were assayed in lean dough. As shown in Fig. 5, the leavening ability of the strains B + T, B + T_-C_, B + T_-B_ and B + T_-A_ increased by 6, 6, 5 and 9%, respectively, compared with the parental strain BY14α. The leavening abilities of the strains B-T + T_-C_, B-T + T_-B_ and B-T + T_-A_, which showed relatively less increases, were 17, 16 and 15% higher than that in the mutant B-TUP1, respectively. Nevertheless, the strain B + T_-E_ showed a 9% decrease in the leavening ability, compared with the strain BY14α. The leavening abilities of the strains B-T + T_-D_ and B-T + T_-E_ were 4 and 6% higher than that of the mutant B-TUP1, but 8 and 6% lower than that of the strain BY14α, respectively. The control strains BYK and B-T + K exhibited similar leavening ability to the strains BY14α and B-TUP1, respectively.

These results reveal that with the native copy of *TUP1* present, overexpression of intact *TUP1* and variant *TUP1* with the Ssn6 interaction, N-terminal repression or C-terminal repression domain truncated were effective for improving the leavening ability of baker’s yeast in lean dough. However, overexpressing the three-domain fragment of *TUP1* negatively affected the fermentation characteristic of baker’s yeast. With *TUP1* deleted from the chromosome, decreased leavening ability resulting from *TUP1* deletion could be significantly countered by overexpression of *TUP1* variants with any of the three domains truncated, but overexpressing *TUP1* with all three domains truncated or the three-domain fragment of *TUP1* did not.

### *MAL62* and *MAL61* expression levels

**Fig. 6:**
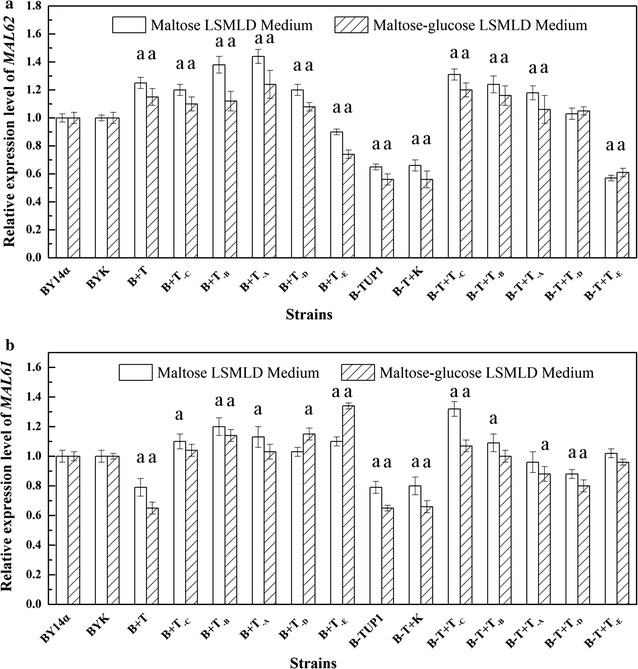
Expression levels of *MAL62* and *MAL61*. Fresh yeast cells were inoculated into LSMLD media and sampled at 1 h. Total RNA was isolated and the expression of **a** *MAL62* and **b** *MAL61* was examined by qRT-PCR. Data are average of three independent experiments and error bars represent ± SD. Significant difference of the strains from the parental strain BY14α was confirmed by Student’s *t* test (^a^ *P* < 0.05, n = 3)

Based on the results regarding maltose utilization and dough leavening, the mRNA levels of *MAL62* encoding for maltase and *MAL61* encoding for maltose permease were quantified. The qRT-PCR results showed that the control strains BYK and B-T + K displayed no significant differences from the strains BY14α and B-TUP1, respectively (Fig. 6). In the strains B + T_-C_, B + T_-B_, B + T_-A_, B + T_-D_, B-T + T_-C_, B-T + T_-B_ and B-T + T_-A_, levels of *MAL62* mRNA increased by 20, 38, 44, 20, 31, 24 and 18% in maltose LSMLD medium, and by 10, 12, 24, 8, 20, 16 and 6% in maltose-glucose LSMLD medium, respectively; *MAL61* mRNA levels influenced to varying degrees, compared with the parental strain BY14α. Compared with the parental strain, the *MAL62* and *MAL61* mRNA levels in the mutant B-TUP1 decreased by 35 and 21% in maltose LSMLD medium, and by 44 and 35% in maltose-glucose LSMLD medium, respectively. Interestingly, the strain B + T increased *MAL62* mRNA level by 25 and 15%, while the *MAL61* mRNA level decreased by 21 and 35%, respectively, in maltose and maltose-glucose media. The changes observed for the strain B + T_-E_ were opposite to that of the strain B + T, with *MAL62* down-regulated by 10 and 26%, and *MAL61* up-regulated by 10 and 34%, respectively, in maltose and maltose-glucose media. In the strain B-T + T_-E_, transcription of *MAL62* was down-regulated by 43 and 39%, in maltose and maltose-glucose media, respectively, and that of *MAL61* was unchanged; but in the strain B-T + T_-D_, the transcription level of *MAL62* was unchanged and that of *MAL61* was down-regulated by 12 and 20%, respectively, in maltose and maltose-glucose media.

These results indicate that deletion of *TUP1* could evidently reduce the expression levels of *MAL62* and *MAL61*. Overexpression of intact *TUP1* and combined overexpression of different domains of Tup1 in different genetic background could affect the *MAL* expression level to varying degrees.

### Growth characteristics

Stable performance of industrial strain is important for fermentation application [24–26]. Thus, we investigated the growth characteristics (specific growth rate and biomass yield) of the strains. As shown in Table 3, the specific growth rate and biomass yield of the strain B + T increased from 0.25 to 0.34 h^−1^ and 6.1 to 6.5 g/L, respectively, compared with the parental strain BY14α. Relative to the parental strain, the strains B + T_-C_ and B + T_-B_ increased the specific growth rate, but did not change the final biomass yield. No obvious changes in specific growth rate and biomass yield were observed in the strains B + T_-A_, B + T_-E_, B + T_-D_, B-TUP1, B-T + T_-C_, B-T + T_-B_, B-T + T_-A_, B-T + T_-E_ and B-T + T_-D_. Similar results were observed for BYK and B-T + K compared to BY14α and B-TUP1, respectively, thus ruling out any possible effects from the transformed empty vector.

**Table 3:** Growth properties of the strains

Strains	Specific growth rate (h^−1^)	Biomass yield (g/L)
BY14α	0.25 ± 0.04	6.1 ± 0.18
BYK	0.24 ± 0.02	6.1 ± 0.16
B + T	0.34 ± 0.06^a^	6.5 ± 0.22^a^
B + T_-C_	0.28 ± 0.04^a^	5.8 ± 0.15
B + T_-B_	0.28 ± 0.05^a^	6.1 ± 0.20
B + T_-A_	0.26 ± 0.03	6.3 ± 0.22
B + T_-D_	0.26 ± 0.04	6.3 ± 0.20
B + T_-E_	0.25 ± 0.03	6.3 ± 0.19
B-TUP1	0.24 ± 0.04	6.1 ± 0.16
B-T + K	0.24 ± 0.03	6.0 ± 0.16
B-T + T_-C_	0.24 ± 0.04	6.1 ± 0.17
B-T + T_-B_	0.25 ± 0.06	6.2 ± 0.20
B-T + T_-A_	0.25 ± 0.05	6.3 ± 0.18
B-T + T_-D_	0.26 ± 0.06	6.3 ± 0.21
B-T + T_-E_	0.25 ± 0.03	6.0 ± 0.14

Values shown represent averages of at least three independent experiments (data are mean ± SD)

^a^Values of the mutants are significantly (Student’s *t* test, *P* < 0.05, n = 3) different from those of the parental strain BY14α

These results show that overexpression of *TUP1* conferred enhanced growth abilities to the baker’s yeast cells. Deletion of *TUP1* and combined overexpression of different domains of Tup1 in different genetic backgrounds did not significantly affect the growth characteristics of baker’s yeast.

## Discussion

In the yeast *S. cerevisiae*, the global transcriptional repressor Tup1 is required for repression of a variety of genes responsible for cellular function. Three functional domains of Tup1 have been identified and are known to have varying effects on transcriptional repression [27]. Herein, we focus on the role of Tup1 and its domains in maltose metabolism of industrial baker’s yeast.

*TUP1* overexpression and deletion have strong but opposing effects on the maltose metabolism and leavening ability of baker’s yeast. The *TUP1* overexpression strain B + T alleviated the glucose repression and increased the *MAL62* expression level. It is probable that Tup1 participated in the positive regulation of the *MAL62* expression through the varying effects of the functional domains in Tup1 or the regulatory Tup1–Ssn6 complex [10]. The effective glucose and *MAL62* derepression by *TUP1* overexpression could facilitate the rapid transition from glucose to maltose metabolism and the efficient maltose hydrolysis, resulting in improved maltose metabolism and dough leavening ability upon *TUP1* overexpression. This finding corresponded with that of Zhang et al. [28], who reported that the enhancing *MAL* expression levels by glucose derepression in *SNF1* (encoding for the catalytic subunit of Snf1 protein kinase, playing a significant role in relieving glucose repression by inactivating the transcription repressor Mig1) overexpression improved the ability of baker’s yeast strain to utilize maltose and leaven lean dough. In addition, overexpression of *TUP1* could enhance the specific growth rate and biomass yield of baker’s yeast. The improvement of growth characteristics was also likely to increase maltose metabolism and leavening ability of *TUP1*-overexpressed strain. Compared with the parental strain, the levels of *MAL61* mRNA decreased upon *TUP1* overexpression, suggesting native negative regulation by the repressor Tup1 [29]. Notably, *TUP1*-overexpressed strain possessing lower *MAL61* level but higher *MAL62* level displayed rapid maltose utilization. This result supports that high level of *MAL62* expression is sufficient to improve maltose metabolism of baker’s yeast [30]. Complete deletion of *TUP1* was negative to glucose and *MAL* derepression levels, thereby resulting in decreased synthesis of proteins involved in maltose utilization [31]. Decreased maltose metabolism with stable physiological characteristic in *TUP1*-deleted mutant was significantly correlated with inferior leavening ability caused by altered *MAL62* and *MAL61* levels, suggesting that *TUP1* was essential to maltose metabolism of baker’s yeast.

It has been well documented that Tup1 contains the Ssn6 interaction, N-terminal repression and C-terminal repression domains [32]. In the presence of the native copy of *TUP1*, strains overexpressing *TUP1* with any of the three domains truncated, i.e. B + T_-C_, B + T_-B_ and B + T_-A_, partially compromised the repression, thus enhancing the maltose metabolism and leavening ability of baker’s yeast in this work. These results support the existence of the three functional domains and suggest their importance in regulating glucose repression by Tup1 [33]. The major cause of the *MAL* derepression is probably that the truncated Tup1 competed with the wild type Tup1 for the formation of the repression complex with Mig1 and Ssn6 to selectively derepress the *MAL* genes, although the complex binds to many other genes as well. The truncated Ssn6 interaction domain of Tup1 in B + T_-A_ could not be recruited by Mig1 to bind to *MAL* promoter. The truncated Tup1 in B + T_-C_ and B + T_-B_ could be recruited to Mig1 for binding *MAL* promoter in truncated Tup1–Ssn6 form, whereas the corepressors could not work properly without repression domains. In contrast, the *MAL62* mRNA level decreased in the strain B + T_-E_. The WD repeat region of Tup1 is required for full Tup1-dependent repression [33]. Therefore, Tup1 containing incomplete WD structure may competitively bind to Mig1 and Ssn6, while weakening interaction with other proteins, presumably elements indirectly involved in regulation of *MAL62* expression (such as proteins involved in sucrose and glucose metabolize), including regulators participated in encoding RNA transcription control and noncoding RNA modification, thereby repressing *MAL62* transcription. Moreover, there is a possibility of synergy between these three domains on the regulation of maltose metabolism. Therefore, overexpression of the three-domain fragment weakened the maltose metabolism and leavening ability of baker’s yeast without changing repression. Compared with the parental strain, no obvious changes in maltose metabolism were observed in the strain B + T_-D_, which overexpressed *TUP1* with the three domains truncated, suggesting the necessity of the Ssn6 interaction, N-terminal repression and C-terminal repression domains and the redundancy of independent truncation of the WD repeats for glucose repression [16, 34].

To better acquaint the effects of different domains within Tup1 on maltose metabolism, different domains of *TUP1* were overexpressed in a *TUP1* deletion mutant. In the *TUP1*-deficient yeast cells, strains overexpressing *TUP1* with either the Ssn6 interaction, N-terminal repression or C-terminal repression domain truncated, i.e. strains B-T + T_-C_, B-T + T_-B_ and B-T + T_-A_, countered the severe repression caused by *TUP1* deletion and generated varying levels of improvement in maltose metabolism and leavening ability. This finding is consistent with the results shown by Lee et al. [18] with respect to galactose metabolism, further suggesting the importance of the Ssn6 interaction, N-terminal repression and C-terminal repression domains for glucose repression. Furthermore, the enhancement in maltose metabolism and leavening ability of the strains B-T + T_-C_, B-T + T_-B_ and B-T + T_-A_ were lower than those in the strains B + T_-C_, B + T_-B_ and B + T_-A_, directly indicating the importance of *TUP1* for maltose metabolism in baker’s yeast. Compared with the parental strain BY14α, the maltose metabolism and leavening ability decreased in the strains B-T + T_-E_ along with diminished *MAL62* level (Fig. 6a) and B-T + T_-D_ with slightly decreased *MAL61* mRNA level (Fig. 6b). It is likely that the first repeat that is missing in the WD region interacts with the Ssn6 interaction, N-terminal repression and C-terminal repression domains or plays roles in other mechanisms affecting the *MAL* genes expression.

## Conclusion

Summarizing the results, the repressor Tup1 is an essential element for regulation of maltose metabolism in industrial baker’s yeast. Importantly, different domains of Tup1 play different roles on glucose repression and maltose metabolism in baker’s yeast cells. The Ssn6 interaction, N-terminal repression and C-terminal repression domains might play important roles in the regulation of *MAL* transcription by Tup1 for maltose metabolism of baker’s yeast. The WD region lacking the first repeat influences the regulation of maltose metabolism directly, rather than indirectly through glucose repression. Although further studies will be needed for clarifying the regulatory mechanism of the different domains in Tup1, these findings lay a foundation for the optimization of industrial baker’s yeast strains for accelerated maltose metabolism and facilitate future research on glucose repression in other sugar metabolism.

## Additional files

**Additional file 1: Figure S1.** Construction process of the plasmid Yep-KPT.

**Additional file 2.** The sequence of plasmid Yep-KPT.

**Additional file 3: Table S1.** Concentration of residual maltose in maltose LSMLD medium (g/L). Concentration of residual maltose of the strains in maltose LSMLD medium at suitable intervals for 4 h.

## References

[1] Hazell B, Attfield P (1999). Enhancement of maltose utilisation by *Saccharomyces cerevisiae* in medium containing fermentable hexoses. J Ind Microbiol Biotechnol.

[2] Needleman R (1991). Control of maltase synthesis in yeast. Mol Microbiol.

[3] Cohen JD, Goldenthal MJ, Chow T, Barbara B, Marmur J (1985). Organization of the *MAL* loci of *Saccharomyces*. Physical identification and functional characterization of three genes at the *MAL6* locus. Mol Gen Genet.

[4] Fagerström-Billai F, Durand-Dubief M, Ekwall K, Wright AP (2007). Individual subunits of the Ssn6-Tup11/12 corepressor are selectively required for repression of different target genes. Mol Cell Biol.

[5] Tanaka N, Mukai Y (2015). Yeast Cyc8p and Tup1p proteins function as coactivators for transcription of Stp1/2p-dependent amino acid transporter genes. Biochem Biophys Res Commun.

[6] Varanasi US, Klis M, Mikesell PB, Trumbly RJ (1996). The Cyc8 (Ssn6)-Tup1 corepressor complex is composed of one Cyc8 and four Tup1 subunits. Mol Cell Biol.

[7] Schachtschabel D, Arentshorst M, Nitsche BM, Morris S, Nielsen KF, van den Hondel CA, Klis FM, Ram AF (2013). The transcriptional repressor TupA in *Aspergillus niger* is involved in controlling gene expression related to cell wall biosynthesis, development, and nitrogen source availability. PLoS ONE.

[8] García-Sánchez S, Mavor AL, Russell CL, Argimon S, Dennison P, Enjalbert B, Brown AJ (2005). Global roles of Ssn6 in Tup1- and Nrg1-dependent gene regulation in the fungal pathogen, *Candida albicans*. Mol Biol Cell..

[9] Jabet C, Sprague ER, VanDemark AP, Wolberger C (2000). Characterization of the N-terminal domain of the yeast transcriptional repressor Tup1. Proposal for an association model of the repressor complex Tup1 x Ssn6. J Biol Chem.

[10] Zhang Z, Reese JC (2004). Redundant mechanisms are used by Ssn6–Tup1 in repressing chromosomal gene transcription in *Saccharomyces cerevisiae*. J Biol Chem.

[11] Redd MJ, Arnaud MB, Johnson AD (1997). A complex composed of tup1 and ssn6 represses transcription in vitro. J Biol Chem.

[12] Edmondson DG, Smith MM, Roth SY (1996). Repression domain of the yeast global repressor Tup1 interacts directly with histones H3 and H4. Genes Dev.

[13] Ferreira ME, Berndt KD, Nilsson J, Wright AP (2010). WD40 domain divergence is important for functional differences between the fission yeast Tup11 and Tup12 co-repressor proteins. PLoS ONE.

[14] Tzamarias D, Struhl K (1994). Functional dissection of the yeast Cyc8-Tup1 transcriptional co-repressor complex. Nature.

[15] Komachi K, Redd MJ, Johnson AD (1994). The WD repeats of Tup1 interact with the homeo domain protein alpha 2. Genes Dev.

[16] Zhang Z, Varanasi U, Trumbly RJ (2002). Functional dissection of the global repressor Tup1 in yeast: dominant role of the C-terminal repression domain. Genetics.

[17] Takayama S, Fujii M, Nakagawa Y, Miki K, Ayusawa D (2011). N-terminal short fragment of *TUP1* confers resistance to 5-bromodeoxyuridine in the yeast *Saccharomyces cerevisiae*. Biochem Biophys Res Commun.

[18] Lee KS, Hong ME, Jung SC, Ha SJ, Yu BJ, Koo HM, Park SM, Seo JH, Kweon DH, Park JC, Jin YS (2011). Improved galactose fermentation of *Saccharomyces cerevisiae* through inverse metabolic engineering. Biotechnol Bioeng.

[19] Lin X, Zhang CY, Bai XW, Song HY, Xiao DG (2014). Effects of *MIG1*, *TUP1* and *SSN6* deletion on maltose metabolism and leavening ability of baker’s yeast in lean dough. Microb Cell Fact.

[20] Zhang CY, Lin X, Song HY, Xiao DG (2015). Effects of *MAL61* and *MAL62* overexpression on maltose fermentation of baker’s yeast in lean dough. World J Microbiol Biotechnol.

[21] Panadero J, Randez-Gil F, Prieto JA (2005). Validation of a flour-free model dough system for throughput studies of baker’s yeast. Appl Environ Microbiol.

[22] Lin X, Zhang CY, Bai XW, Feng B, Xiao DG (2015). Improvement of stress tolerance and leavening ability under multiple baking-associated stress conditions by overexpression of the *SNR84* gene in baker’s yeast. Int J Food Microbiol.

[23] Lin X, Zhang CY, Bai XW, Xiao DG (2015). Effects of *GLC7* and *REG1* deletion on maltose metabolism and leavening ability of baker’s yeast in lean dough. J Biotechnol.

[24] Chen Y, Li F, Guo J, Liu G, Guo X, Xiao D (2014). Enhanced ethyl caproate production of Chinese liquor yeast by overexpressing *EHT1* with deleted *FAA1*. J Ind Microbiol Biotechnol.

[25] Zhang CY, Lin X, Feng B, Liu XE, Bai XW, Xu J, Pi L, Xiao DG (2016). Enhanced leavening properties of baker’s yeast by reducing sucrase activity in sweet dough. Appl Microbiol Biotechnol.

[26] Dong J, Xu H, Zhao L, Chen Y, Zhang C, Guo X, Hou X, Chen D, Zhang C, Xiao D (2014). Enhanced acetate ester production of Chinese liquor yeast by overexpressing *ATF1* through precise and seamless insertion of *PGK1* promoter. J Ind Microbiol Biotechnol.

[27] Green SR, Johnson AD (2005). Genome-wide analysis of the functions of a conserved surface on the corepressor Tup1. Mol Biol Cell.

[28] Zhang CY, Bai XW, Lin X, Liu XE, Xiao DG (2015). Effects of *SNF1* on maltose metabolism and leavening ability of baker’s yeast in lean dough. J Food Sci.

[29] Parnell EJ, Stillman DJ (2011). Shields up: the Tup1-Cyc8 repressor complex blocks coactivator recruitment. Genes Dev.

[30] Sun X, Zhang C, Dong J, Wu M, Zhang Y, Xiao D (2012). Enhanced leavening properties of baker’s yeast overexpressing *MAL62* with deletion of *MIG1* in lean dough. J Ind Microbiol Biotechnol.

[31] Hu Z, Nehlin JO, Ronne H, Michels CA (1995). *MIG1*-dependent and *MIG1*-independent glucose regulation of *MAL* gene expression in *Saccharomyces cerevisiae*. Curr Genet.

[32] Lamas-Maceiras M, Freire-Picos MA, Torres AM (2011). Transcriptional repression by *Kluyveromyces lactis* Tup1 in *Saccharomyces cerevisiae*. J Ind Microbiol Biotechnol.

[33] Carrico PM, Zitomer RS (1998). Mutational analysis of the Tup1 general repressor of yeast. Genetics.

[34] Zhang Z, Varanasi U, Carrico P, Trumbly RJ (2002). Mutations of the WD repeats that compromise Tup1 repression function maintain structural integrity of the WD domain trypsin-resistant core. Arch Biochem Biophys.

