# Supplementary figures and images for: Functional analysis of the global repressor Tup1 for maltose metabolism in Saccharomyces cerevisiae: different roles of the functional domains
Source: Microb Cell Fact. 2017 Nov 9;16:194. doi: 10.1186/s12934-017-0806-6 (PMC5679332; doi:10.1186/s12934-017-0806-6)

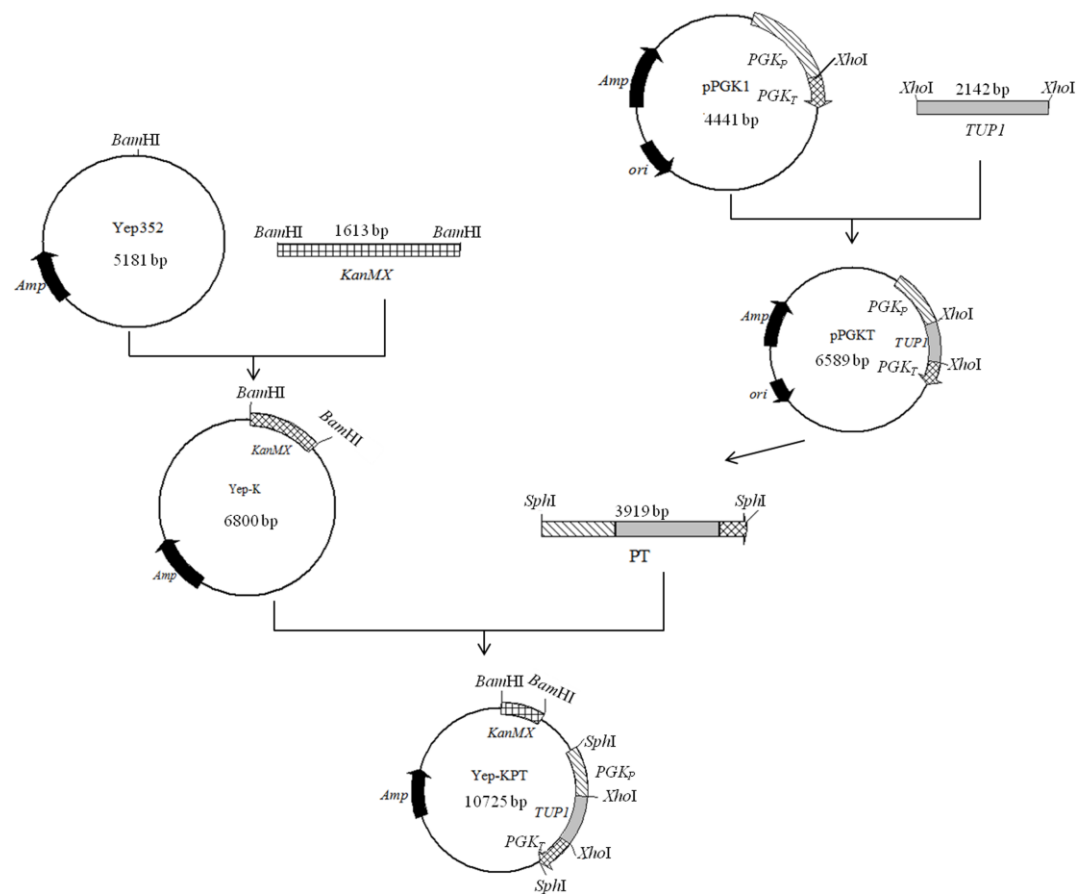

Fig. S1 Construction process of the plasmid Yep-KPT.

Supplement: Supplementary file 1 — Additional file 1: Figure S1. Construction process of the plasmid Yep-KPT. [file 12934_2017_806_MOESM1_ESM.pdf]
