# Supplementary material for: Functional analysis of the global repressor Tup1 for maltose metabolism in Saccharomyces cerevisiae: different roles of the functional domains
Source: Microb Cell Fact. 2017 Nov 9;16:194. doi: 10.1186/s12934-017-0806-6 (PMC5679332; doi:10.1186/s12934-017-0806-6)
Supplement: Supplementary file 2 — Additional file 2. The sequence of plasmid Yep-KPT. [file 12934_2017_806_MOESM2_ESM.pdf]

The sequence of plasmid Yep-KPT (Yep-KanMX-PGK1-TUP1) is as followed (Restriction sites are underlined. *KanMX* sequence is in green. The sequence of *PGK1* promoter is in blue. *TUP1* sequence is in pink. The sequence of *PGK1* terminator is in orange):

1 ATGACCATGA TTACGAATTC GAGCTCGGTA CCCGGGGATC CCAGCTGAAG CTTCGTACGC
61 TGCAGGTCGA CAACCCTTAA TATAACTTCG TATAATGTAT GCTATACGAA GTTATTAGGT
121 CTAGAGATCT GTTTAGCTTG CCTCGTCCCC GCCGGGTCAC CCGGCCAGCG ACATGGAGGC
181 CCAGAATACC CTCCTTGACA GTCTTGACGT GCGCAGCTCA GGGGCATGAT GTGACTGTCG
241 CCCGTACATT TAGCCCATAC ATCCCCATGT ATAATCATTT GCATCCATAC ATTTTGATGG
301 CCGCACGGCG CGAAGCAAAA ATTACGGCTC CTCGCTGCAG ACCTGCGAGC AGGGAAACGC
361 TCCCCTCACA GACGCGTTGA ATTGTCCCCA CGCCGCGCCC CTGTAGAGAA ATATAAAAGG
421 TTAGGATTTG CCACTGAGGT TCTTCTTTCA TATACTTCCT TTTAAAATCT TGCTAGGATA
481 CAGTTCTCAC ATCACATCCG AACATAAACA ACCATGGGTA AGGAAAAGAC TCACGTTTCG
541 AGGCCGCGAT TAAATTCCAA CATGGATGCT GATTTATATG GGTATAAATG GGCTCGCGAT
601 AATGTCGGGC AATCAGGTGC GACAATCTAT CGATTGTATG GGAAGCCCGA TGCGCCAGAG
661 TTGTTTCTGA AACATGGCAA AGGTAGCGTT GCCAATGATG TTACAGATGA GATGGTCAGA
721 CTAAACTGGC TGACGGAATT TATGCCTCTT CCGACCATCA AGCATTTTAT CCGTACTCCT
781 GATGATGCAT GGTTACTCAC CACTGCGATC CCCGGCAAAA CAGCATTCCA GGTATTAGAA
841 GAATATCCTG ATTCAGGTGA AAATATTGTT GATGCGCTGG CAGTGTTCCT GCGCCGGTTG
901 CATTCGATTC CTGTTTGTAA TTGTCCTTTT AACAGCGATC GCGTATTTCG TCTCGCTCAG
961 GCGCAATCAC GAATGAATAA CGGTTTGGTT GATGCGAGTG ATTTTGATGA CGAGCGTAAT
1021 GGCTGGCCTG TTGAACAAGT CTGGAAAGAA ATGCATAAGC TTTTGCCATT CTCACCGGAT
1081 TCAGTCGTCA CTCATGGTGA TTTCTCACTT GATAACCTTA TTTTTGACGA GGGGAAATTA
1141 ATAGGTTGTA TTGATGTTGG ACGAGTCGGA ATCGCAGACC GATACCAGGA TCTTGCCATC
1201 CTATGGAACT GCCTCGGTGA GTTTTCTCCT TCATTACAGA AACGGCTTTT TCAAAAATAT
1261 GGTATTGATA ATCCTGATAT GAATAAATTG CAGTTTCATT TGATGCTCGA TGAGTTTTTC
1321 TAATCAGTAC TGACAATAAA AAGATTCTTG TTTTCAAGAA CTTGTCATTT GTATAGTTTT
1381 TTTATATTGT AGTTGTTCTA TTTTAATCAA ATGTTAGCGT GATTTATATT TTTTTTCGCC
1441 TCGACATCAT CTGCCCAGAT GCGAAGTTAA GTGCGCAGAA AGTAATATCA TGCGTCAATC
1501 GTATGTGAAT GCTGGTCGCT ATACTGCTGT CGATTCGATA CTAACGCCGC CATCCAGTGT
1561 CGAAAACGAG CTCTCGAGAA CCCTTAATAT AACTTCGTAT AATGTATGCT ATACGAAGTT
1621 ATTAGGTGAT ATCAGATCCA CTAGTGGCCT ATGCGGATCC TCTAGAGTCG ACCTGCAGGC
1681 ATGCTCTAAC TGATCTATCC AAAACTGAAA ATTACATTCT TGATTAGGTT TATCACAGGC
1741 AAATGTAATT TGTGGTATTT TGCCGTTCAA AATCTGTAGA ATTTTCTCAT TGGTCACATT
1801 ACAACCTGAA AATACTTTAT CTACAATCAT ACCATTCTTA TAACATGTCC CCTTAATACT
1861 AGGATCAGGC ATGAACGCAT CACAGACAAA ATCTTCTTGA CAAACGTCAC AATTGATCCC
1921 TCCCCATCCG TTATCACAAT GACAGGTGTC ATTTTGTGCT CTTATGGGAC GATCCTTATT
1981 ACCGCTTTCA TCCGGTGATA GACCGCCACA GAGGGGCAGA GAGCAATCAT CACCTGCAAA
2041 CCCTTCTATA CACTCACATC TACCAGTGTA CGAATTGCAT TCAGAAAACT GTTTGCATTC
2101 AAAAATAGGT AGCATACAAT TAAAACATGG CGGGCATGTA TCATTGCCCT TATCTTGTGC

2161 AGTTAGACGC GAATTTTTCG AAGAAGTACC TTCAAAGAAT GGGGTCTTAT CTTGTTTTGC
2221 AAGTACCACT GAGCAGGATA ATAATAGAAA TGATAATATA CTATAGTAGA GATAACGTCG
2281 ATGACTTCCC ATACTGTAAT TGCTTTTAGT TGTGTATTTT TAGTGTGCAA GTTTCTGTAA
2341 ATCGATTAAT TTTTTTTTCT TTCCTCTTTT TATTAACCTT AATTTTTATT TTAGATTCCT
2401 GACTTCAACT CAAGACGCAC AGATATTATA ACATCTGCAT AATAGGCATT TGCAAGAATT
2461 ACTCGTGAGT AAGGAAAGAG TGAGGAACTA TCGCATACCT GCATTTAAAG ATGCCGATTT
2521 GGGCGCGAAT CCTTTATTTT GGCTTCACCC TCATACTATT ATCAGGGCCA GAAAAAGGAA
2581 GTGTTTCCCT CCTTCTTGAA TTGATGTTAC CCTCATAAAG CACGTGGCCT CTTATCGAGA
2641 AAGAAATTAC CGTCGCTCGT GATTTGTTTG CAAAAAGAAC AAAACTGAAA AAACCCAGAC
2701 ACGCTCGACT TCCTGTCTTC CTATTGATTG CAGCTTCCAA TTTCGTCACA CAACAAGGTC
2761 CTAGCGACGG CTCACAGGTT TTGTAACAAG CAATCGAAGG TTCTGGAATG GCGGGAAAGG
2821 GTTTAGTACC ACATGCTATG ATGCCCACTG TGATCTCCAG AGCAAAGTTC GTTCGATCGT
2881 ACTGTTACTC TCTCTCTTTC AAACAGAATT GTCCGAATCG TGTGACAACA ACAGCCTGTT
2941 CTCACACACT CTTTTCTTCT AACCAAGGGG GTGGTTTAGT TTAGTAGAAC CTCGTGAAAC
3001 TTACATTTAC ATATATATAA ACTTGCATAA ATTGGTCAAT GCAAGAAATA CATATTTGGT
3061 CTTTTCTAAT TCGTAGTTTT TCAAGTTCTT AGATGCTTTC TTTTTCTCTT TTTTACAGAT
3121 CATCAAGGAA GTAATTATCT ACTTTTTACA ACAAATATAA AACAAGATCG GAATTCCAGA
3181 TCTCCTCGAG ATGACTGCCA GCGTTTCGAA TACGCAGAAT AAGCTGAATG AGCTTCTCGA
3241 TGCCATCAGA CAGGAGTTTC TCCAAGTCTC ACAAGAGGCA AATACCTACC GTCTTCAAAA
3301 CCAAAAGGAT TACGATTTCA AAATGAACCA GCAGCTGGCT GAGATGCAGC AGATAAGAAA
3361 CACCGTCTAC GAACTGGAAC TAACTCACAG GAAAATGAAG GACGCGTACG AAGAAGAGAT
3421 CAAGCACTTG AAACTAGGGC TGGAGCAAAG AGACCATCAA ATTGCATCTT TGACCGTCCA
3481 GCAACAGCGG CAACAGCAAC AGCAGCAACA GGTCCAGCAG CATTTACAAC AGCAACAGCA
3541 GCAGCTAGCC GCTGCATCTG CATCTGTTCC AGTTGCGCAA CAACCACCGG CTACTACTTC
3601 GGCCACCGCC ACTCCAGCAG CAAACACAAC TACTGGTTCG CCATCGGCCT TCCCAGTACA
3661 AGCTAGCCGT CCTAATCTGG TTGGCTCACA GTTGCCTACC ACCACTTTGC CTGTGGTGTC
3721 CTCAAACGCC CAACAACAAC TACCACAACA GCAACTGCAA CAGCAGCAAC TTCAACAACA
3781 GCAACCACCT CCCCAGGTTT CCGTGGCACC ATTGAGTAAC ACAGCCATCA ACGGATCTCC
3841 TACTTCTAAA GAGACCACTA CTTTACCCTC TGTCAAGGCA CCTGAATCTA CGTTGAAAGA
3901 AACTGAACCG GAAAATAATA ATACCTCGAA GATAAATGAC ACCGGATCCG CCACCACGGC
3961 CACCACTACC ACCGCAACTG AAACTGAAAT CAAACCTAAG GAGGAAGACG CCACCCCGGC
4021 TAGTTTGCAC CAGGATCACT ACTTAGTCCC TTATAATCAA AGAGCAAACC ACTCTAAACC
4081 TATCCCACCT TTCCTTTTGG ATCTAGATTC CCAGTCTGTT CCCGATGCTC TGAAGAAGCA
4141 AACAAATGAT TATTATATTT TATACAACCC GGCACTACCA AGAGAAATTG ACGTTGAGTT
4201 ACACAAATCT TTGGATCATA CTTCAGTTGT TTGTTGCGTG AAGTTCAGTA ACGATGGTGA
4261 ATACTTAGCC ACAGGCTGCA ACAAAACTAC TCAAGTGTAT CGCGTTTCAG ATGGTTCTCT
4321 GGTGGCCCGT CTATCTGACG ATTCTGCTGC CAATAACCAT CGAAATTCGA TCACTGAAAA
4381 TAACACCACC ACGTCCACGG ATAACAATAC AATGACAACC ACTACTACCA CCACAATTAC
4441 TACCACAGCG ATGACTTCGG CAGCAGAATT GGCAAAAGAT GTGGAAAACC TGAACACTTC
4501 GTCTTCCCCA TCATCCGACT TGTATATCCG TTCAGTGTGT TTTTCTCCAG ATGGGAAATT
4561 TTTGGCAACA GGTGCTGAAG ACAGACTGAT TAGAATTTGG GATATTGAAA ATAGAAAGAT
4621 TGTTATGATT CTTCAAGGCC ACGAACAAGA TATTTATTCA TTGGACTACT TTCCCTCAGG
4681 TGACAAATTA GTCTCCGGTT CTGGTGACCG TACCGTTCGT ATTTGGGACT TACGTACAGG
4741 CCAGTGTTCA TTGACTTTAT CCATTGAAGA TGGTGTTACC ACCGTCGCTG TATCACCAGG

4801 TGATGGTAAA TACATCGCTG CTGGTTCTCT AGATCGTGCT GTGAGAGTTT GGGATTCCGA
4861 GACCGGATTC TTGGTGGAAA GACTAGATTC GGAAAACGAA TCCGGTACAG GCCACAAGGA
4921 CTCTGTTTAT AGCGTTGTCT TCACTAGAGA TGGACAAAGC GTTGTATCCG GCTCATTAGA
4981 TAGATCTGTT AAGCTCTGGA ATTTGCAGAA TGCAAACAAC AAGAGCGATT CGAAAACTCC
5041 AAATTCCGGC ACTTGTGAAG TTACGTATAT CGGGCATAAA GACTTTGTAT TGTCCGTGGC
5101 CACCACACAA AATGATGAGT ACATCTTGTC CGGTTCCAAA GATCGTGGTG TCCTGTTTTG
5161 GGATAAGAAA TCCGGCAATC CGTTATTGAT GTTGCAAGGT CATAGGAATT CAGTTATATC
5221 TGTGGCTGTG GCAAACGGGT CTCCGCTGGG TCCAGAATAT AACGTTTTTG CTACTGGTAG
5281 CGGTGATTGT AAAGCAAGGA TTTGGAAGTA TAAAAAAATA GCGCCAAATT AACTCGAGGG
5341 ATCTGCGATA GATCAATTTT TTTCTTTTCT CTTTCCCCAT CCTTTACGCT AAAATAATAG
5401 TTTATTTTAT TTTTTGAATA TTTTTTATTT ATATACGTAT ATATAGACTA TTATTTATCT
5461 TTTAATGATT ATTAAGATTT TTATTAAAAA AAAATTCGCT CCTCTTTTAA TGCCTTTATG
5521 CAGTTTTTTT TTCCCATTCG ATATTTCTAT GTTCGGGTTC AGCGTATTTT AAGTTTAATA
5581 ACTCGAAAAT TCTGCGTTCG TTAGCATGCA AGCTTGGCAC TGGCCGTCGT TTTACAACGT
5641 CGTGACTGGG AAAACCCTGG CGTTACCCAA CTTAATCGCC TTGCAGCACA TCCCCCCTTC
5701 GCCAGCTGGC GTAATAGCGA AGAGGCCCGC ACCGATCGCC CTTCCCAACA GTTGCGCAGC
5761 CTGAATGGCG AATGGCGCCT GATGCGGTAT TTTCTCCTTA CGCATCTGTG CGGTATTTCA
5821 CACCGCATAG GGTAATAACT GATATAATTA AATTGAAGCT CTAATTTGTG AGTTTAGTAT
5881 ACATGCATTT ACTTATAATA CAGTTTTTTA GTTTTGCTGG CCGCATCTTC TCAAATATGC
5941 TTCCCAGCCT GCTTTTCTGT AACGTTCACC CTCTACCTTA GCATCCCTTC CCTTTGCAAA
6001 TAGTCCTCTT CCAACAATAA TAATGTCAGA TCCTGTAGAG ACCACATCAT CCACGGTTCT
6061 ATACTGTTGA CCCAATGCGT CTCCCTTGTC ATCTAAACCC ACACCGGGTG TCATAATCAA
6121 CCAATCGTAA CCTTCATCTC TTCCACCCAT GTCTCTTTGA GCAATAAAGC CGATAACAAA
6181 ATCTTTGTCG CTCTTCGCAA TGTCAACAGT ACCCTTAGTA TATTCTCCAG TAGATAGGGA
6241 GCCCTTGCAT GACAATTCTG CTAACATCAA AAGGCCTCTA GGTTCCTTTG TTACTTCTTC
6301 TGCCGCCTGC TTCAAACCGC TAACAATACC TGGGCCCACC ACACCGTGTG CATTCGTAAT
6361 GTCTGCCCAT TCTGCTATTC TGTATACACC CGCAGAGTAC TGCAATTTGA CTGTATTACC
6421 AATGTCAGCA AATTTTCTGT CTTCGAAGAG TAAAAAATTG TACTTGGCGG ATAATGCCTT
6481 TAGCGGCTTA ACTGTGCCCT CCATGGAAAA ATCAGTCAAG ATATCCACAT GTGTTTTTAG
6541 TAAACAAATT TTGGGACCTA ATGCTTCAAC TAACTCCAGT AATTCCTTGG TGGTACGAAC
6601 ATCCAATGAA GCACACAAGT TTGTTTGCTT TTCGTGCATG ATATTAAATA GCTTGGCAGC
6661 AACAGGACTA GGATGAGTAG CAGCACGTTC CTTATATGTA GCTTTCGACA TGATTTATCT
6721 TCGTTTCGGT TTTTGTTCTG TGCAGTTGGG TTAAGAATAC TGGGCAATTT CATGTTTCTT
6781 CAACACTACA TATGCGTATA TATACCAATC TAAGTCTGTG CTCCTTCCTT CGTTCTTCCT
6841 TCTGTTCGGA GATTACCGAA TCAAAAAAAT TTCAAAGAAA CCGAAATCAA AAAAAAGAAT
6901 AAAAAAAAAA TGATGAATTG AAAAGCTCTT GTTACCCATC ATTGAATTTT GAACATCCGA
6961 ACCTGGGAGT TTTCCCTGAA ACAGATAGTA TATTTGAACC TGTATAATAA TATATAGTCT
7021 AGCGCTTTAC GGAAGACAAT GTATGTATTT CGGTTCCTGG AGAAACTATT GCATCTATTG
7081 CATAGGTAAT CTTGCACGTC GCATCCCCGG TTCATTTTCT GCGTTTCCAT CTTGCACTTC
7141 AATAGCATAT CTTTGTTAAC GAAGCATCTG TGCTTCATTT TGTAGAACAA AAATGCAACG
7201 CGAGAGCGCT AATTTTTCAA ACAAAGAATC TGAGCTGCAT TTTTACAGAA CAGAAATGCA
7261 ACGCGAAAGC GCTATTTTAC CAACGAAGAA TCTGTGCTTC ATTTTTGTAA AACAAAAATG
7321 CAACGCGAGA GCGCTAATTT TTCAAACAAA GAATCTGAGC TGCATTTTTA CAGAACAGAA
7381 ATGCAACGCG AGAGCGCTAT TTTACCAACA AAGAATCTAT ACTTCTTTTT TGTTCTACAA

7441 AAATGCATCC CGAGAGCGCT ATTTTTCTAA CAAAGCATCT TAGATTACTT TTTTTCTCCT
7501 TTGTGCGCTC TATAATGCAG TCTCTTGATA ACTTTTTGCA CTGTAGGTCC GTTAAGGTTA
7561 GAAGAAGGCT ACTTTGGTGT CTATTTTCTC TTCCATAAAA AAAGCCTGAC TCCACTTCCC
7621 GCGTTTACTG ATTACTAGCG AAGCTGCGGG TGCATTTTTT CAAGATAAAG GCATCCCCGA
7681 TTATATTCTA TACCGATGTG GATTGCGCAT ACTTTGTGAA CAGAAAGTGA TAGCGTTGAT
7741 GATTCTTCAT TGGTCAGAAA ATTATGAACG GTTTCTTCTA TTTTGTCTCT ATATACTACG
7801 TATAGGAAAT GTTTACATTT TCGTATTGTT TTCGATTCAC TCTATGAATA GTTCTTACTA
7861 CAATTTTTTT GTCTAAAGAG TAATACTAGA GATAAACATA AAAAATGTAG AGGTCGAGTT
7921 TAGATGCAAG TTCAAGGAGC GAAAGGTGGA TGGGTAGGTT ATATAGGGAT ATAGCACAGA
7981 GATATATAGC AAAGAGATAC TTTTGAGCAA TGTTTGTGGA AGCGGTATTC GCAATATTTT
8041 AGTAGCTCGT TACAGTCCGG TGCGTTTTTG GTTTTTTGAA AGTGCGTCTT CAGAGCGCTT
8101 TTGGTTTTCA AAAGCGCTCT GAAGTTCCTA TACTTTCTAG CTAGAGAATA GGAACTTCGG
8161 AATAGGAACT TCAAAGCGTT TCCGAAAACG AGCGCTTCCG AAAATGCAAC GCGAGCTGCG
8221 CACATACAGC TCACTGTTCA CGTCGCACCT ATATCTGCGT GTTGCCTGTA TATATATATA
8281 CATGAGAAGA ACGGCATAGT GCGTGTTTAT GCTTAAATGC GTTATGGTGC ACTCTCAGTA
8341 CAATCTGCTC TGATGCCGCA TAGTTAAGCC AGCCCCGACA CCCGCCAACA CCCGCTGACG
8401 CGCCCTGACG GGCTTGTCTG CTCCCGGCAT CCGCTTACAG ACAAGCTGTG ACCGTCTCCG
8461 GGAGCTGCAT GTGTCAGAGG TTTTCACCGT CATCACCGAA ACGCGCGAGA CGAAAGGGCC
8521 TCGTGATACG CCTATTTTTA TAGGTTAATG TCATGATAAT AATGGTTTCT TAGACGTCAG
8581 GTGGCACTTT TCGGGGAAAT GTGCGCGGAA CCCCTATTTG TTTATTTTTC TAAATACATT
8641 CAAATATGTA TCCGCTCATG AGACAATAAC CCTGATAAAT GCTTCAATAA TATTGAAAAA
8701 GGAAGAGTAT GAGTATTCAA CATTTCCGTG TCGCCCTTAT TCCCTTTTTT GCGGCATTTT
8761 GCCTTCCTGT TTTTGCTCAC CCAGAAACGC TGGTGAAAGT AAAAGATGCT GAAGATCAGT
8821 TGGGTGCACG AGTGGGTTAC ATCGAACTGG ATCTCAACAG CGGTAAGATC CTTGAGAGTT
8881 TTCGCCCCGA AGAACGTTTT CCAATGATGA GCACTTTTAA AGTTCTGCTA TGTGGCGCGG
8941 TATTATCCCG TATTGACGCC GGGCAAGAGC AACTCGGTCG CCGCATACAC TATTCTCAGA
9001 ATGACTTGGT TGAGTACTCA CCAGTCACAG AAAAGCATCT TACGGATGGC ATGACAGTAA
9061 GAGAATTATG CAGTGCTGCC ATAACCATGA GTGATAACAC TGCGGCCAAC TTACTTCTGA
9121 CAACGATCGG AGGACCGAAG GAGCTAACCG CTTTTTTGCA CAACATGGGG GATCATGTAA
9181 CTCGCCTTGA TCGTTGGGAA CCGGAGCTGA ATGAAGCCAT ACCAAACGAC GAGCGTGACA
9241 CCACGATGCC TGTAGCAATG GCAACAACGT TGCGCAAACT ATTAACTGGC GAACTACTTA
9301 CTCTAGCTTC CCGGCAACAA TTAATAGACT GGATGGAGGC GGATAAAGTT GCAGGACCAC
9361 TTCTGCGCTC GGCCCTTCCG GCTGGCTGGT TTATTGCTGA TAAATCTGGA GCCGGTGAGC
9421 GTGGGTCTCG CGGTATCATT GCAGCACTGG GGCCAGATGG TAAGCCCTCC CGTATCGTAG
9481 TTATCTACAC GACGGGGAGT CAGGCAACTA TGGATGAACG AAATAGACAG ATCGCTGAGA
9541 TAGGTGCCTC ACTGATTAAG CATTGGTAAC TGTCAGACCA AGTTTACTCA TATATACTTT
9601 AGATTGATTT AAAACTTCAT TTTTAATTTA AAAGGATCTA GGTGAAGATC CTTTTTGATA
9661 ATCTCATGAC CAAAATCCCT TAACGTGAGT TTTCGTTCCA CTGAGCGTCA GACCCCGTAG
9721 AAAAGATCAA AGGATCTTCT TGAGATCCTT TTTTTCTGCG CGTAATCTGC TGCTTGCAAA
9781 CAAAAAAACC ACCGCTACCA GCGGTGGTTT GTTTGCCGGA TCAAGAGCTA CCAACTCTTT
9841 TTCCGAAGGT AACTGGCTTC AGCAGAGCGC AGATACCAAA TACTGTCCTT CTAGTGTAGC
9901 CGTAGTTAGG CCACCACTTC AAGAACTCTG TAGCACCGCC TACATACCTC GCTCTGCTAA
9961 TCCTGTTACC AGTGGCTGCT GCCAGTGGCG ATAAGTCGTG TCTTACCGGG TTGGACTCAA
10021 GACGATAGTT ACCGGATAAG GCGCAGCGGT CGGGCTGAAC GGGGGGTTCG TGCACACAGC

10081 CCAGCTTGGA GCGAACGACC TACACCGAAC TGAGATACCT ACAGCGTGAG CATTGAGAAA
10141 GCGCCACGCT TCCCGAAGGG AGAAAGGCGG ACAGGTATCC GGTAAGCGGC AGGGTCGGAA
10201 CAGGAGAGCG CACGAGGGAG CTTCCAGGGG GAAACGCCTG GTATCTTTAT AGTCCTGTCG
10261 GGTTTCGCCA CCTCTGACTT GAGCGTCGAT TTTTGTGATG CTCGTCAGGG GGGCGGAGCC
10321 TATGGAAAAA CGCCAGCAAC GCGGCCTTTT TACGGTTCCT GGCCTTTTGC TGGCCTTTTG
10381 CTCACATGTT CTTTCCTGCG TTATCCCCTG ATTCTGTGGA TAACCGTATT ACCGCCTTTG
10441 AGTGAGCTGA TACCGCTCGC CGCAGCCGAA CGACCGAGCG CAGCGAGTCA GTGAGCGAGG
10501 AAGCGGAAGA GCGCCCAATA CGCAAACCGC CTCTCCCCGC GCGTTGGCCG ATTCATTAAT
10561 CCAGCTGGCA CGACAGGTTT CCCGACTGGA AAGCGGGCAG TGAGCGCAAC GCAATTAATG
10621 TGAGTTACCT CACTCATTAG GCACCCCAGG CTTTACACTT TATGCTTCCG GCTCGTATGT
10681 TGTGTGGAAT TGTGAGCGGA TAACAATTTC ACACAGGAAA CAGCT
