# Supplementary material for: Functional analysis of the global repressor Tup1 for maltose metabolism in Saccharomyces cerevisiae: different roles of the functional domains
Source: Microb Cell Fact. 2017 Nov 9;16:194. doi: 10.1186/s12934-017-0806-6 (PMC5679332; doi:10.1186/s12934-017-0806-6)
Supplement: Supplementary file 3 — Additional file 3: Table S1. Concentration of residual maltose in maltose LSMLD medium (g/L). Concentration of residual maltose of the strains in maltose LSMLD medium at suitable intervals for 4 h. [file 12934_2017_806_MOESM3_ESM.pdf]

Table S1 Concentration of residual maltose in maltose LSMLD medium (g/L)

Strains \ Fermentation time (min)	0	30	60	90	120	150	180	240
BY14α	37.83 ± 0.89	36.50 ± 0.78	32.33 ± 0.66	28.60 ± 0.62	25.34 ± 0.45	20.05 ± 0.40	13.93 ± 0.38	6.19 ± 0.26
BYK	37. 86 ± 0.90	36.53 ± 0.67	32.53 ± 0.54	29.02 ± 0.58	25.12 ± 0.50	19.87 ± 0.44	13.76 ± 0.25	6.17 ± 0.21
B+T	38.02 ± 0.78	36.28 ± 0.72	32.05 ± 0.60	28.34 ± 0.51	23.92 ± 0.44	19.37 ± 0.35	12.47 ± 0.30	5.68 ± 0.12[a]
B+T$_{-C}$	38.00 ± 0.89	36.34 ± 0.78	32.14 ± 0.64	28.46 ± 0.60	25.26 ± 0.48	19.47 ± 0.41	11.76 ± 0.30	5.71 ± 0.16[a]
B+T$_{-B}$	37.68 ± 0.74	36.67 ± 0.70	32.45 ± 0.65	28.12 ± 0.55	25.10 ± 0.50	19.86 ± 0.44	13.69 ± 0.32	6.03 ± 0.26
B+T$_{-A}$	38.12 ± 0.65	36.30 ± 0.61	32.78 ± 0.60	28.83 ± 0.60	25.06 ± 0.51	20.00 ± 0.50	13.40 ± 0.44	5.97 ± 0.15
B+T$_{-D}$	37.91 ± 0.78	35.51 ± 0.70	33.60 ± 0.62	28.86 ± 0.60	24.99 ± 0.55	20.72 ± 0.46	12.84 ± 0.40	6.18 ± 0.20
B+T$_{-E}$	38.10 ± 0.80	36.63 ± 0.74	34.17 ± 0.68	28.83 ± 0.64	24.45 ± 0.58	20.19 ± 0.50	14.44 ± 0.50	7.34 ± 0.30[a]
B-TUP1	38. 00 ± 0.92	36.69 ± 0.77	32.91 ± 0.64	27.95 ± 0.58	26.00 ± 0.54	20.83 ± 0.42	15.73 ± 0.40	6.88 ± 0.22[a]
B-T+K	38.02 ± 0.88	36.58 ± 0.72	32.65 ± 0.60	28.24 ± 0.60	25.92 ± 0.44	20.37 ± 0.35	15.47 ± 0.30	6.78 ± 0. 18[a]
B-T+T$_{-C}$	38.10 ± 0.89	35.77 ± 0.75	32.07 ± 0.60	27.04 ± 0.57	22.62 ± 0.40	18.31 ± 0.41	12.91 ± 0.27	5.71 ± 0.16[ab]
B-T+T$_{-B}$	38.20 ± 0.90	35.63 ± 0.70	31.87 ± 0.65	26.58 ± 0.53	23.34 ± 0.50	19.49 ± 0.41	12.84 ± 0.22	5.64 ± 0.19[ab]
B-T+T$_{-A}$	37.69 ± 0.71	35.19 ± 0.63	31.29 ± 0.63	27.08 ± 0.60	23.55 ± 0.55	19.21 ± 0.51	12.53 ± 0.32	5.46 ± 0.15[ab]
B-T+T$_{-D}$	38.15 ± 0.78	35.54 ± 0.72	33.17 ± 0.62	28.33 ± 0.60	24.65 ± 0.48	19.69 ± 0.45	14.47 ± 0.36	6.44 ± 0.21[b]
B-T+T$_{-E}$	37.90 ± 0.80	35.66 ± 0.74	32.71 ± 0.70	28.79 ± 0.65	24.74 ± 0.55	20.88 ± 0.50	14.38 ± 0.40	6.33 ± 0.20[b]

Values shown represent averages of at least three independent experiments (data are means ± SD).

[a] Values of the mutants are significantly (Student's *t* test, $P < 0.05$, n=3) different from that of the parental strain BY14α.

[b] Values of the mutants are significantly (Student's *t* test, $P < 0.05$, n=3) different from that of the mutant B-TUP1.
